# Participatory research involving people with neurodegenerative diseases and family caregivers: a systematic review

**DOI:** 10.1002/alz.091205

**Published:** 2025-01-09

**Authors:** Marcel Daum, Fenia Gkolfo Ferra, Eva Drewelow, Peggy Walde, Kai Gerullis, Ingo Kilimann, Birgit Völlm, Stefan Teipel, Olga A. Klein

**Affiliations:** ^1^ Rostock University Medical Center, Rostock Germany; ^2^ University Medical Center Rostock, Rostock Germany; ^3^ Rostock University Medical Center, 18147 Germany; ^4^ Department of Psychosomatic Medicine, Rostock University Medical Center, Rostock Germany; ^5^ German Center for Neurodegenerative Diseases (DZNE), Rostock Germany; ^6^ University of Rostock, Rostock Germany; ^7^ Deutsches Zentrum für Neurodegenerative Erkrankungen e. V. (DZNE), Rostock Germany; ^8^ University Medical Center Rostock, 18147 Germany

## Abstract

**Background:**

Participatory research or patient and public involvement refer to the process of actively involving people with lived experience into the research process to improve its relevance, quality, and impact. In the PART project we aim to establish a sustainable structure to include underrepresented patient groups with neurodegenerative diseases into a patient advisory board for research. As one of our milestones, we conducted a systematic literature review with the aim of examining the impact of participatory research on people involved, such as those with cognitive impairment, caregivers, and researchers.

**Objective:**

To explore the benefits, limitations and barriers of participatory research in the field of neurodegenerative diseases.

**Method:**

We conducted a systematic literature review covering the databases PubMed, PsycINFO, CINAHL, Embase, Scopus, ClinicalTrials.gov, and Google Scholar, and added hand searches. The search strategy consisted of the terms “patients” AND “dementia” AND “participation” AND “impact” AND “research”. Based on the selection of relevant articles published between 2000 and 2022, we performed a quality assessment using the Mixed Methods Appraisal Tool (MMAT).

**Result:**

The entire search yielded 1,994 articles, of which 1,169 remained after the removal of duplicates and were integrated into the screening process. Following a two‐step screening process and subsequent validation of a subset of 20% of the articles by a second reviewer, a set of 30 articles were selected for inclusion in the review. Current findings indicate that participatory research in the field of neurodegenerative diseases provides various advantages, such as empowering people with lived experience and enhancing researchers' understanding of those affected. Challenges included the extra time and effort required, as well as the difficulty of establishing a balanced relationship between researchers and co‐researchers.

**Conclusion:**

The number and quality of scientific articles regarding participatory research in the field of neurodegenerative diseases has been growing over the past two decades. The terminology used in reporting was frequently ambiguous, the description of participatory methods was often insufficient, and the impacts were frequently disregarded. Further research is required to address these issues and to investigate the impact of peer research in dementia studies.

